# METTL1 promotes neuroblastoma development through m^7^G tRNA modification and selective oncogenic gene translation

**DOI:** 10.1186/s40364-022-00414-z

**Published:** 2022-09-07

**Authors:** Ying Huang, Jieyi Ma, Cuiyun Yang, Paijia Wei, Minghui Yang, Hui Han, Hua Dong Chen, Tianfang Yue, Shu Xiao, Xuanyu Chen, Zuoqing Li, Yanlai Tang, Jiesi Luo, Shuibin Lin, Libin Huang

**Affiliations:** 1 Department of Pediatrics, The First Affiliated Hospital, Sun Yat-sen University, Guangzhou, 510080 China; 2 Center for Translational Medicine, Precision Medicine Institute, The First Affiliated Hospital, Sun Yat-sen University, Guangzhou, 510080 China; 3 Department of Clinical Laboratory, The Second Affiliated Hospital of Guangzhou University of Chinese Medicine, Guangzhou, People’s Republic of China; 4 Department of Pediatric Surgery, The First Affiliated Hospital, Sun Yat-sen University, Guangzhou, 510080 China

**Keywords:** Neuroblastoma, N7-methylguanosine, Epigenetics

## Abstract

**Background:**

Neuroblastoma (NBL) is the most common extra-cranial solid tumour in childhood, with prognosis ranging from spontaneous remission to high risk for rapid and fatal progression. Despite existing therapy approaches, the 5-year event-free survival (EFS) for patients with advanced NBL remains below 30%, emphasizing urgent necessary for novel therapeutic strategies. Studies have shown that epigenetic disorders play an essential role in the pathogenesis of NBL. However, the function and mechanism of N7-methylguanosine (m^7^G) methyltransferase in NBL remains unknown.

**Methods:**

The expression levels of m^7^G tRNA methyltransferase Methyltransferase-like 1 (METTL1) were analyzed by querying the Gene Expression Omnibus (GEO) database and further confirmed by immunohistochemistry (IHC) assay. Kaplan-Meier, univariate and multivariate cox hazard analysis were performed to reveal the prognostic role of METTL1. Cell function assays were performed to evaluate how METTL1 works in proliferation, apoptosis and migration in cell lines and xenograft mouse models. The role of METTL1 on mRNA translation activity of NBL cells was measured using puromycin intake assay and polysome profiling assay. The m^7^G modified tRNAs were identified by tRNA reduction and cleavage sequencing (TRAC-seq). Ribosome nascent-chain complex-bound mRNA sequencing (RNC-seq) was utilized to identify the variation of gene translation efficiency (TE). Analyzed the codon frequency decoded by m^7^G tRNA to clarify the translation regulation and mechanism of m^7^G modification in NBL.

**Results:**

This study found that METTL1 were significantly up-regulated in advanced NBL, which acted as an independent risk factor and predicted poor prognosis. Further in NBL cell lines and BALB/c-nu female mice, we found METTL1 played a crucial role in promoting NBL progression. Furthermore, m^7^G profiling and translation analysis revealed downregulation of METTL1 would inhibit puromycin intake efficiency of NBL cells, indicating that METTL1 did count crucially in regulation of NBL cell translation. With all tRNAs with m^7^G modification identified in NBL cells, knockdown of METTL1 would significantly reduce the levels of both m^7^G modification and m^7^G tRNAs expressions. Result of RNC-seq shew there were 339 overlapped genes with impaired translation in NBL cells upon METTL1 knockdown. Further analysis revealed these genes contained higher frequency of codons decoded by m^7^G-modified tRNAs and were enriched in oncogenic pathways.

**Conclusion:**

This study revealed the critical role and mechanism of METTL1-mediated tRNA m^7^G modification in regulating NBL progression, providing new insights for developing therapeutic approaches for NBL patients.

**Supplementary Information:**

The online version contains supplementary material available at 10.1186/s40364-022-00414-z.

## Background

Neuroblastoma (NBL) is the most common malignant tumor in infancy and the most common extracranial solid tumor in childhood, accounting for more than 7% of malignant tumors in children under 15 years of age [1]. Despite the availability of multimodal treatment, survival for advanced NBL are extremely low. It poses many challenges for clinicians in diagnosis and treatment, especially for high risk NBL [1, 2]. Therefore, it is vital to search for potential molecular mechanisms of NBL initiation and progression as well as more effective management strategies.

Epigenetic disorders have been proven to play an essential role in the pathogenesis of NBL and offer a number of potential therapeutic targets [3–8]. In recent years, with the continuous optimization of the high-throughput sequencing technologies, a large number of low-abundance RNA modifications on mRNA, tRNA, and other non-coding RNA (ncRNA) have been shown to be associated with the onset and progression of human diseases [9–12]. A growing number of reports show that aberrant expression of long non-coding RNAs (lncRNAs), dysregulated expression and functional disruption of microRNAs (miRNAs) serve a crucial function for explaining the expression of MYCN proto-oncogene (MYCN) and the malignant progression in high risk NBL [13–18]. In addition, N6-methyladenosine (m^6^A) mRNA modification regulators have been reported to affect the NBL prognosis and may be the novel therapeutic targets for NBL [19]. Indeed, tRNAs contain more modifications that contribute to tRNA stability, translation accuracy, and protein synthesis rates [20, 21]. Aberrant expression or mutations of tRNA modification enzymes are increasingly observed in human diseases [22, 23]. However, little is known about the physiological functions of tRNA modifications, especially in cancers.

tRNAs are widely modified in nature, and N7-methylguanosine (m^7^G) is one of the most prevalent modified nucleosides [24]. This modification is performed by m^7^G methyltransferase, which in humans is installed by the METTL1 (methyltransferase-like 1) /WDR4 (WD repeat domain 4) complex [25, 26]. The complex component METTL1 catalyzes methylation of guanine, while its partner WDR4 helps stabilize the methyltransferase complex [25, 27]. Recent reports have revealed a widespread tRNA m^7^G methylome in mammals [26, 28–30]. Knockout of METTL1 leads to impaired tRNA m^7^G modification and abnormal differentiation and growth of embryonic stem cells in mice [26]. Mutations in WDR4 are associated with microcephalic primordial dwarfism and Galloway-Mowat syndrome [31, 32]. These evidences suggest that METTL1/WDR4-mediated tRNA m^7^G modifications play a key role in regulating cell fate decisions. Notably, METTL1 was recently reported to impair the sensitivity of colon and cervical cancer cells to chemotherapy [33]. Nevertheless, the oncogenic functions and molecular mechanisms of m^7^G tRNA modification in regulating NBL progression remain uncovered.

Here, we found that evaluated METTL1 was an independent risk factor for patients with NBL and predicts poor prognosis. Functionally, knockdown of METTL1 was attributed to weakened the capability of tumorigenesis and progression in vitro and in vivo. Mechanistically, METTL1-mediated tRNA m^7^G modification selectively regulates the translation of oncogenic transcripts in a codon frequency-dependent manner. This study revealed the molecular mechanism by which tRNA m^7^G modification mediated NBL progression, providing a potential strategy for clinical management of NBL.

## Materials and methods

### Patient samples

Clinical data were obtained from 132 children (under 16 years old) with NBL diagnosed pathologically as neuroblastoma (with INSS stage4S cases removed) at the First Affiliated Hospital of Sun Yat-sen University from January 1, 2014, to December 30, 2019. Clinical data were applied to analyze the relationship between the diagnostic stage and METTL1 expression levels. Paraffin-embedded specimens were used for immunohistochemistry (IHC) analysis of METTL1 expression levels. This project was approved by the Medical Ethical committee for Clinical Research and Animal Trails of the First Affiliated Hospital of Sun Yat-sen University (Application ID:[2020]486). Baseline information of NBL sample were listed in Supplementary Table 1.

The NBL datasets (GSE62564, https://www.ncbi.nlm.nih.gov/geo/query/acc.cgi?acc=GSE62564) from GEO database were used for the univariate and multivariate cox hazard analysis of risk factors of NBL and the analysis of association between the expression of METTL1 and NBL patients’ prognosis. Baesline information of GSE62564 were listed in Supplementary Table 2.

### Experimental animals

The BALB/c-nu female mice were purchased from GemPharmatech Co., Ltd. (Jiangsu, China). All animal care and experimental protocols were approved by the Institutional Ethics Committee for Clinical Research and Animal Trials of the First Affiliated Hospital of Sun Yat-sen University. The study complied with all relevant ethical regulations regarding Animal Research: Reporting of in vivo Experiments (ARRIVE) guidelines. Mice were euthanized when their tumor size and overall health status met the institutional euthanasia criteria. This project was approved by the Medical Ethical committee for Clinical Research and Animal Trails of the First Affiliated Hospital of Sun Yat-sen University (Application ID:[2020]486).

### Cell lines and cultures

Human embryonic kidney 293 T (HEK 293 T) cells were obtained from Prof. Shuibin Lin’s laboratory (Guangzhou, China). Human NBL KELLY cells were obtained from Tongpai Biotechnology Co Ltd. (Shanghai, China). Human NBL SK-N-BE (2) C (BE2C) cells were from Procell Life Science & Technology Ltd. (Wuhan, China). 293 T cells were cultured in Dulbecco’s Modified Eagle Medium (DMEM, Gibco, USA) supplemented with 10% fetal bovine serum (FBS, Gibco, USA) and 1% penicillin-streptomycin (Gibco, USA), and 1% GlutaMAX (Gibco, USA). KELLY cells were cultured in DMEM (Gibco, USA) supplemented with 10% FBS (Gibco, Australia) and 1% penicillin-streptomycin (Gibco, USA), and 1% GlutaMAX (Gibco, USA). BE2C cells were cultured in Dulbecco’s Modified Eagle Medium/Nutrient Mixture F-12 (DMEM/F-12, Gibco, USA) supplemented with 10% FBS (Gibco, Australia), 1% penicillin-streptomycin (Gibco, USA), 1× GlutaMAX (Gibco, USA), and 1% MEM non-essential amino acid solution (Gibco, USA). Cells were cultured in a 5% CO_2_ cell culture incubator (Thermo Scientific, USA) at 37 °C.

### Knockdown of METTL1 in NBL cells

Lentiviral vectors expressing pLKO.1 Short hairpin RNA (shRNA) as a negative control and two shRNA constructs targeting METTL1 were supplied by KeyGEN BioTECH (Nanjing, China). For lentivirus production, the lentiviral shRNA constructs were co-transfected into 293 T cells using Lipofectamine 2000 (Invitrogen, USA) with a packaging plasmid pCMV-ΔR8.9 and an envelope plasmid pCMV-VSVG. 48 hours later, the viruses were collected and infected with Polybrene (Solarbio, China) (4 μg/ml for KELLY cells and 8 μg/ml for BE2C cells). Stably infected cells were screened with puromycin (Solarbio, China) (2 μg/ml for KEELY cells and 4 μg/ml for BE2C cells) for 24 hours. Small interfering RNA (siRNA) targeting the 3′ untranslated regions (3′ UTR) of METTL1 were used to knockdown METTL1 with Lipofectamine 2000 (Invitrogen, USA). siRNA sequences are listed in Supplementary Table 3.

### Cell proliferation and migration assays

For the cell proliferation assay, 1000 cells were grown in each well of a 96-well plate with 100 μL of fresh medium. Cell viabilities were measured every 24 hours for five days using Cell Counting Kit-8 (Dojindo, Japan). For the migration assay, 7.5 × 10^4^ cells in 200 μL of serum-free medium were added to the upper chamber of the transwell insert (Corning Falcon, USA) and placed in receiving wells containing 700 μL of cell culture medium supplemented with 10% FBS. Migrated cells were stained with 0.5% crystal violet and counted after 24 hours.

### Cell apoptosis assays

According to the manufacturer’s instructions, the cell apoptosis assay was performed with Annexin V-FITC Apoptosis Detection Kit (KeyGEN BioTECH, China). The percentage of positive cells was detected by CytoFLEX (Beckman Coulter, USA).

### Subcutaneous implantation in a mouse model

Four to six-week-old BALB/c-nu female mice were randomly divided into two groups: shRNA targeting green fluorescent protein (shGFP) (*N* = 5) and shRNA targeting METTL1–1 (shM1–1) (N = 5). NBL cells were resuspended by mixing equal amounts of phosphate-buffered saline (PBS, Gibco, USA) and Matrigel (Corning, China). 7× 10^6^ NBL cells in 100 μl of the PBS-Matrigel mixture were injected into the back of the mice. The length (a) and width (b) of the tumors were measured every two days with calipers, and the tumor volume (V) was calculated using the formula V = ab^2^/2. Fifteen days after injection, the mice were humanely killed, and the harvested tumors were used for further analysis.

### Immunohistochemical (IHC) staining

IHC was performed with an IHC kit (Agilent, USA) and anti-METTL1 antibody (Proteintech, 1:2000 dilution) and anti-Ki67 antibody (Proteintech, 1:8000 dilution), to detect the described protein expression. To assessing the level of METTL1 expression, histochemistry score (H-score) is generating by the following formula: H-Score = summation (i × pi)，where i is the intensity score and pi is the percent of the cells with that intensity. The intensity score was categorized as 0 (absent), 1 (weak), 2 (moderate) and 3 (strong). The percent of the cells was scored as 0, 1, 2, 3 and 4 for < 5, 5 to 25%, 25 to 50%, 50 to 75%, and > 75%, respectively. Tissues with H-score≧6 (the median H-score) were considered as high METTL1 expression group, and tissues with H-score < 6 were classified as low METTL1 expression group. The antibodies used in this study are listed in Supplementary Table 4.

### RNA isolation and quantitative analysis

According to the manufacturer’s instructions, total RNAs were isolated with AG RNAex Pro RNA Reagent (AG, China). For reverse transcription-polymerase chain reaction (RT-PCR), cDNA was synthesized in a 20uL reaction system using HiScript III RT SuperMix for qPCR Kit (Vazyme, China). cDNA samples were then diluted at 1:20 and used for Real-time quantitative PCR assays (qPCR). qPCR were performed on a StepOnePlus™ real-time PCR system (Thermo Scientific, USA) with TB Green™ Premix Ex Taq™ II (Takara, Japan). Each sample was repeated three times. Results were calculated by the 2^-ΔΔCt^ method using β-ACTIN primer as an internal control. The primer sequences used in this study are listed in Supplementary Table 3.

### Northern blot, northwestern blot, and Western blot

As previously reported, northern blot and western blot assays were performed [26, 34]. Briefly, for northern blot, 2 μg of total RNA was separated by electrophoresis on a 15% TBE-UREA gel containing tris base, boric acid, Ethylene Diamine Tetraacetic Acid (EDTA) and urea. Then the RNAs were transferred to a positively charged nylon membrane. Afterward, the nylon membranes were cross-linked with ultraviolet light. The indicated tRNAs and U6 small nuclear RNA (snRNA) were blotted with Digoxigenin-labeled probes. After transfer and cross-linking, nylon membranes were blotted with primary antibody, anti-m^7^G antibody (MBL International, 1:1000 dilution), for Northwestern blotting at 4 °C overnight. Anti-Digoxigenin-AP (Roche 1:15000 dilution), or anti-m^7^G antibody signals were detected according to the previously described Western blot protocol [26, 34]. Probe sequences are listed in Supplementary Table 3. The antibodies used in this study are listed in Supplementary Table 4.

### Polysome profiling

Polysome profiling was performed as previously described [35]. Briefly, NBL cells were incubated with 100 μg/ml cycloheximide for 15 minutes at 37 °C. After immediate cold PBS wash, the cells were lysed with multimeric cell extraction buffer containing 50 mM 3- (N-morpholino) propanesulfonic acid (MOPS), 15 mM MgCl2, 150 mM NaCl, 100 μg/ml cycloheximide, 0.5% Triton X-100, 1 mg/ml heparin, 200 U/ml RNase inhibitor, 2 mM Phenylmethylsulfonyl Fluoride (PMSF), and 1 mM benzamidine for 10 minutes on ice and centrifuged at 13,000 g for 10 minutes at 4 °C. The optical density (OD) values of the samples were measured and adjusted to be equal. Then 1 ml of cytoplasmic extract was layered onto 11 ml of a 10–50% sucrose gradient, followed by centrifugation at 36,000 rpm for 3 hours at 4 °C. Separated samples were fractionated at 0.75 ml/minutes by a BR-188 density gradient fractionation system (Brandel, USA) and monitored at an absorbance of 254 nm.

### Puromycin intake assay

NBL cells were transfected with siRNA targeting METTL1 (siM1) and siRNA targeting negative control oligos (siNC). After 48 hours, cells were incubated by using puromycin with final concentration of 1 μM for 30 minutes at 37 °C. After incubation, the cells were lysed to extract proteins, and the levels of puromycin were detected by Western blot with an anti-puromycin antibody (Millipore, 1:2000 dilution). The siRNA sequences are listed in Supplementary Table 3. The antibodies used in this study are listed in Supplementary Table 4.

### tRNA m^7^G reduction and cleavage sequencing (TRAC-seq)

TRAC-seq was performed as previously described [36]. Small RNAs were isolated from total RNAs using the Quick-RNA™ Microprep Kit (Zymo Research, USA) according to the manufacturer’s instructions, followed by recombinant wild-type and D135S AlkB protein treatment. Half of the AlkB-treated RNAs were used as input for the construction of the library. Next, the remaining AlkB-treated RNAs were treated with 0.2 M NaBH4 for 30 minutes on ice in the dark. The RNAs were then dark-treated with aniline acetate solution (H2O: glacial acetic acid: aniline, 7:3:1) for 2 h at room temperature in the dark to induce the site-specific cleavage. After the cleavage, the RNAs were purified using the Oligo Clean & Concentrator™ kit (Zymo Research, USA). Finally, the RNA samples were applied for cDNA library construction using NEBNext Multiplex Small RNA Library Prep Set for Illumina (New England BioLabs, USA) and used for high-throughput sequencing on Illumina Nextseq 500. The TRAC-seq data were analyzed as previously described [36]. Briefly, for tRNA m^7^G analysis, joint and low-quality sequence data were filtered with Trim-Galore. The filtered data were mapped to human mature tRNA sequences using Bowtie, and the read depth at each site and the number of reads starting from that position were calculated using Bedtools. Chemical treatment (NaBH4/aniline-treated) in TRAC-Seq resulted in cleavage of tRNA at m^7^G site, which were detected by sequencing. Therefore, the principle of tRNA m^7^G site recognition is founded on cleavage sites. In order to obtain the global m^7^G cleavage sites in tRNAs, the cleavage scores were determined by comparing the ratio of reads starting at the specific site to reads passing through that site in treated and non-treated (control samples without NaBH4/aniline treatment) samples. Then, the cleavage ratio of site i is defined as the ratio between the number of reads starting and the read depth at the site i. And the cleavage score of the site was then calculated as:

$$\mathrm{Cleavage}\;\mathrm{score}\;\left(\mathrm i\right)=\frac{\log\;2\;\left({\mathrm{Cleavage}\;\mathrm{ratio}}_{\mathrm{treat}}\right)}{\log\;2\;\left({\mathrm{Cleavage}\;\mathrm{ratio}}_{\mathrm{non}-\mathrm{treat}}\right)}$$

Sites with a cleavage score > 3 and a cleavage rate > 0.1 were considered as candidate m^7^G sites. To analyze tRNA expression, we extracted sequences containing tRNA genes and 100 bp upstream and downstream of tRNA genes as precursor tRNA genes. The predicted introns were deleted for the mature tRNA sequences, and “CCA” was added to the 3′ end. During the mapping process with Bowtie2, tRNA reads were calculated and normalized for further analysis.

### Ribosome nascent-chain complex-bound mRNA sequencing (RNC-seq)

RNC-seq was performed as previously described [37]. Briefly, cells were pretreated with 100 μg/ml cycloheximide and incubated for 15 minutes at 37 °C. After washing twice with pre-cooled PBS, 1 ml of cell lysate was incubated with 1 ml of ribosomal buffer (RB buffer) containing 1% TritonX-100 [20 mM HEPES-KOH (pH 7.4), 15 mM MgCl_2_, 200 mM KCl, 100 μg/ml cycloheximide and 2 mM dithiothreitol] for 30 minutes on ice. The cell lysate was then centrifuged at 16,200 g for 10 minutes at 4 °C. 10% of the extract was used as input control. The remaining extract was layered into 11.5 ml sucrose buffer (30% sucrose in RB buffer), and the RNC pellet was collected by centrifugation at 32,000 rpm for 5 h at 4 °C. Next, RNA was isolated from the input and RNC samples for sequencing. The isolated RNA was subjected to cDNA library construction and sequencing using the BGISEQ-500 platform (BGI-Shenzhen, China). Gene expression levels were normalized to FPKM (Fragments per kilobase per million). Translational efficiencies were calculated as: TE = (FPKM in polyribosome-seq) / (FPKM in input RNA-seq).

### Gene ontology and pathway analysis

Gene ontology and pathway analysis of TE-down (Down-regulated Translational efficiencies) mRNAs identified in RNC-seq data were performed using ToppGene Webtool (https://toppgene.cchmc.org/enrichment.jsp). Benjamini-Hochberg adjusted *P* values < 0.05 for ontology terms, and pathways were classified as significantly enriched.

### Statistics analysis

Quantitative data are shown as mean ± SEM. *P values* in all cases are represented as: ****P < 0.0001, ***p < 0.001, **p < 0.01, *p < 0.05. For statistical analysis, Student’s t-test, one-way ANOVA, or Mann-Whitney U test were used unless otherwise stated. Event-free survival was analyzed using the log-rank test. Statistical analyses were performed using R studio, GraphPad Prism version 8 and SPSS version 25.

## Results

### Elevated METTL1 is associated with poor prognosis in NBL patients

**Fig. 1:**
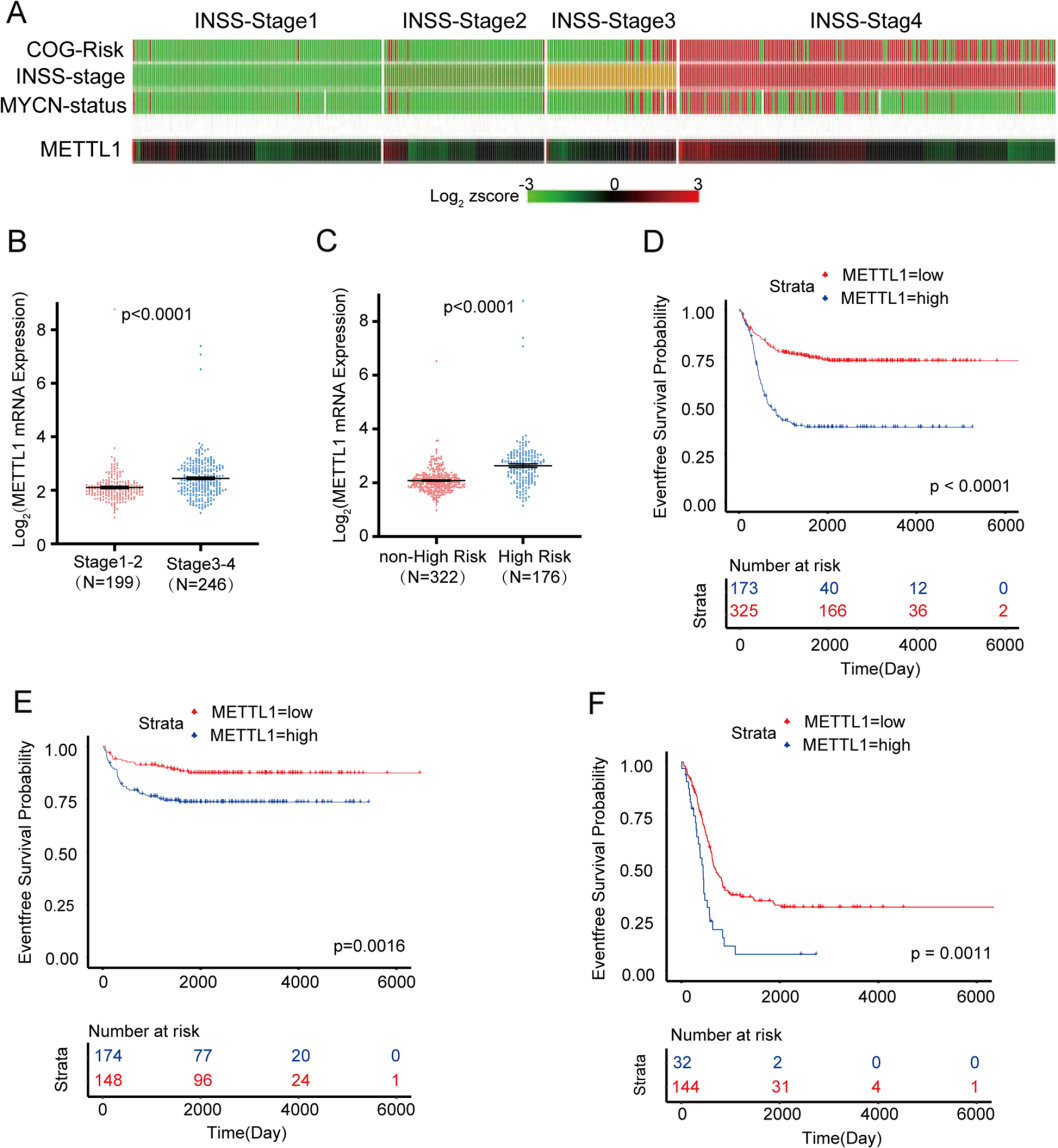
METTL1 is elevated in advanced NBLs and is associated with poor prognosis in NBL patients in the GEO database (GSE62564). **A** The R2 genomics analysis and visualization platform showed a heatmap of correlations between METTL1 mRNA levels, COG risk, INSS stage, and MYCN status in NBL samples from the GEO database. **B** The GEO database showed the mRNA level of METTL1 in NBL samples at different stages (except stage4S, *N* = 53). **C** GEO database showed METTL1 mRNA levels in high-risk NBL samples and non-high-risk NBL samples. **D** Kaplan-Meier analysis of the event-free survival of patients grouped by METTL1 expression (*N* = 498). **E**, **F** Event-free survival based on METTL1 expression in the non-high risk cohort (**E**, *N* = 322) and high risk cohort (**F**, *N* = 176). Data are presented as Mean ± SEM. *P* values were calculated by two-tailed Student’s test for (**B**, **C**) and Log-rank test for (**D**-**F**)

**Fig. 2:**
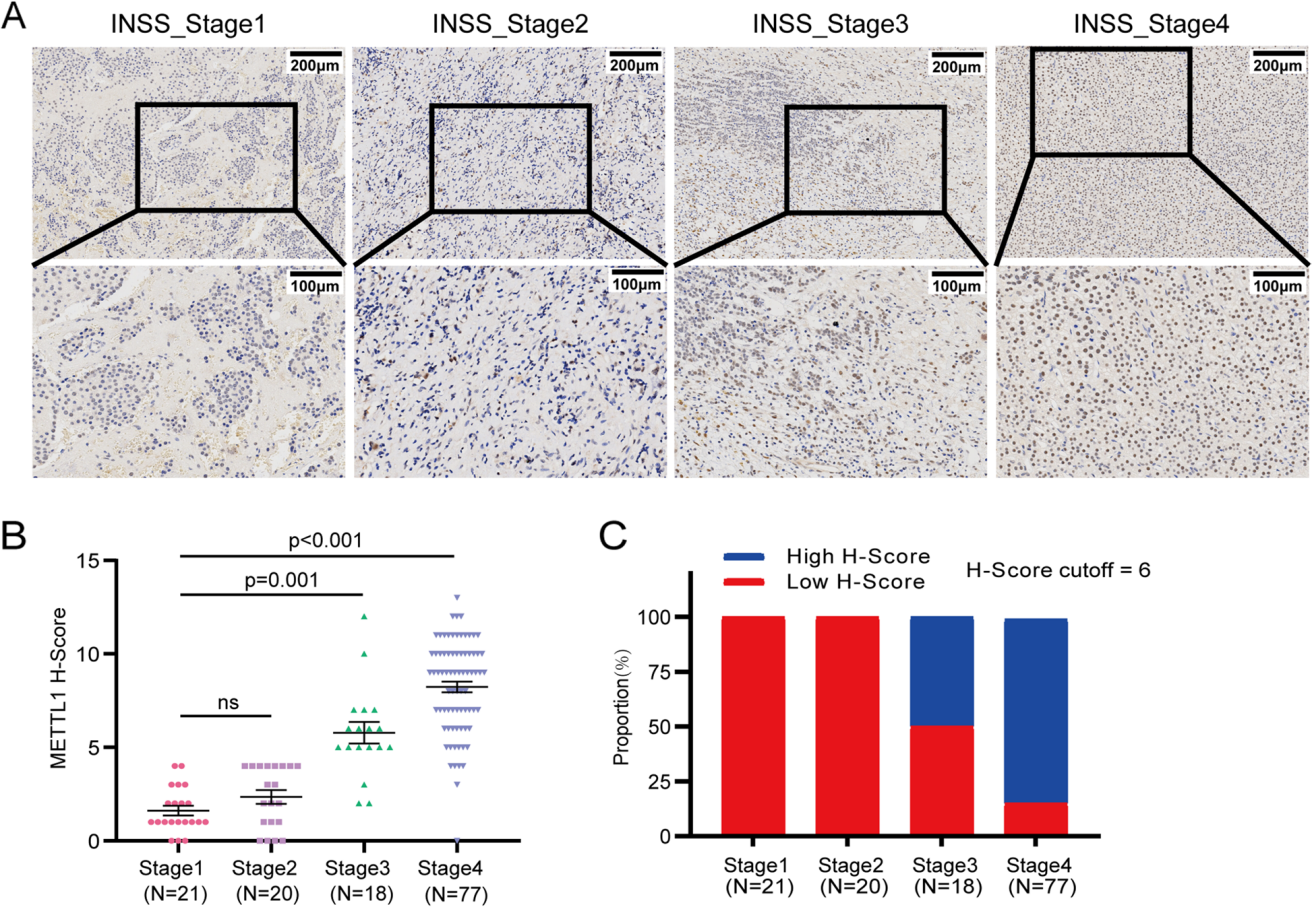
METTL1 is elevated in advanced NBLs and is associated with poor prognosis in patients with clinical NBL. **A** Representative images of METTL1 IHC staining with different staining intensities in clinical NBL tumors. **B** Quantification of METTL1 IHC scores in different INSS stages of clinical NBL tumors. **C** Proportion of METTL1expression cases at different stages of clinical NBL tumors. Data are presented as Mean ± SEM*. P* values were calculated by Kruskal-Wallis test with Dunnett’s multiple comparisons test

To explore the clinical relation of METTL1 and NBL, we first analyzed the RNA-seq and corresponding clinical information of NBL from the GEO database (GSE62564). We found that METTL1 highly expressed in high risk NBL based on the Children’s Oncology Group (COG), advanced NBL according to the International Neuroblastoma Staging System (INSS), and NBL with MYCN amplification (Fig. 1A). In addition, METTL1 mRNA levels were significantly elevated in the advanced INSS stage cohort and the high risk cohort (Fig. 1B, C). Further Kaplan-Meier analysis showed that NBL patients with low METTL1 expression had a significantly better 5-year EFS than those with high METTL1 expression (Fig. 1D and Supplementary Fig. 1A, cutoff = 5.09, *p* < 0.001). We next analyzed 493 of the 498 patients in a retrospective cohort study, and 5 cases were excluded for lack of MYCN status records. Both univariate (Supplementary Table 5) and multivariate (Supplementary Fig. 1B and Supplementary Table 5) Cox regression analysis of EFS from diagnosis revealed that METTL1 was the independent risk factor beyond INSS stage, age, MYCN status and COG risk classification (HR = 1.58, %95CI 1.09 to 2.29, *p* = 0.016). Moreover, the group with high levels of METTL1 had a worse prognosis in non-high risk group (Fig. 1E and Supplementary Fig. 1C, cutoff = 3.99, *p* = 0.0016) and high risk group (Fig. 1F and Supplementary Fig. 1D, cutoff = 8.46, *p* = 0.0011) respectively, suggesting a potential regulatory role in the progression of NBL. Similarily, with (Supplementary Fig. 1E-F) or without (Supplementary Fig. 1G, H) MYCN amplification, patients with high levels of METTL1 exhibited worse EFS (without MYCN amplification: cutoff = 4.92, *p* < 0,001; with MYCN amplification: cutoff = 9.06, *p* = 0.023) than patients with low levels of METTL1. To further validate the potential link between METTL1 and NBL, METTL1 protein levels were examined by IHC in a cohort of 132 NBL patients (Fig. 2A). IHC was assessed by H-score. The results consistently showed that METTL1 protein levels were significantly increased in advanced NBL (Fig. 2B, C), which further demonstrated that high METTL1 expression was closely associated with the high INSS stage of NBL. Our findings suggested that METTL1 could play an essential role in the progression of NBL.

### METTL1 knockdown inhibited NBL progression in vitro

**Fig. 3:**
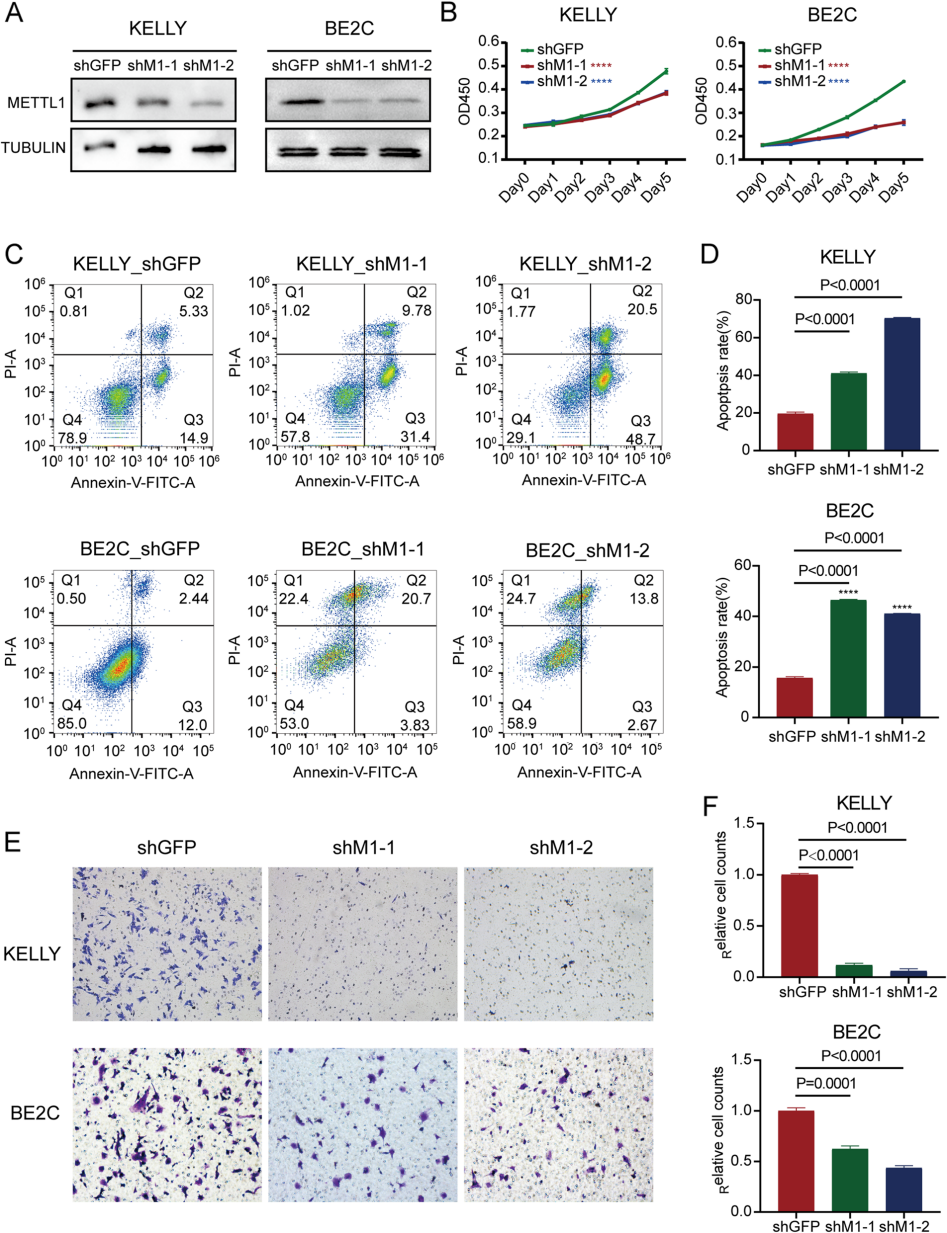
METTL1 knockdown inhibits NBL progression in vitro. **A** Western blot confirmed stable knockdown of METTL1 in KELLY and BE2C cells. **B** The CCK-8 assay (*N* = 3 per group) determined the proliferation of METTL1 knockdown and control NBL cells. **C**-**D** Flow cytometry assay (**C**) and quantitative analysis (**D**) of apoptosis rates in METTL1 knockdown and control NBL cells. **E**-**F** Migration assay (**E**) and quantitative analysis (**F**) of METTL1 knockdown and control NBL cells. Data are presented as Mean ± SEM. *P* values were calculated by One-way ANOVA with Dunnett’s multiple comparisons test

To explore the role of METTL1 in NBL, shM1–1 and shM1–2 were used to knockdown the expression of METTL1. KELLY and BE2C were two NBL cell lines with high METTL1 expression. Cells transfected with shGFP were used as the negative control. Western blot assays were performed to confirm the inhibitory effect of METTL1 in KELLY and BE2C cells (Fig. 3A). To further study the biological effects of METTL1 knockdown in NBL cells, we first performed a CCK-8 assay to test the proliferation of NBL cells. The results showed that downregulation of METTL1 significantly inhibited cell proliferation (Fig. 3B). In addition, flow cytometry assays were performed, and the data showed that knockdown of METTL1 would result in a significant increase in apoptosis of NBL cells (Fig. 3C, D). Furthermore, the migration ability of NBL cells was significantly decreased after METTL1 knockdown compared to the control group (Fig. 3E, F). These results uncovered the critical role of METTL1 in the progression of NBL in vitro.

### Inhibition of METTL1 reduced the tumorigenicity of NBL cells in vivo

**Fig. 4:**
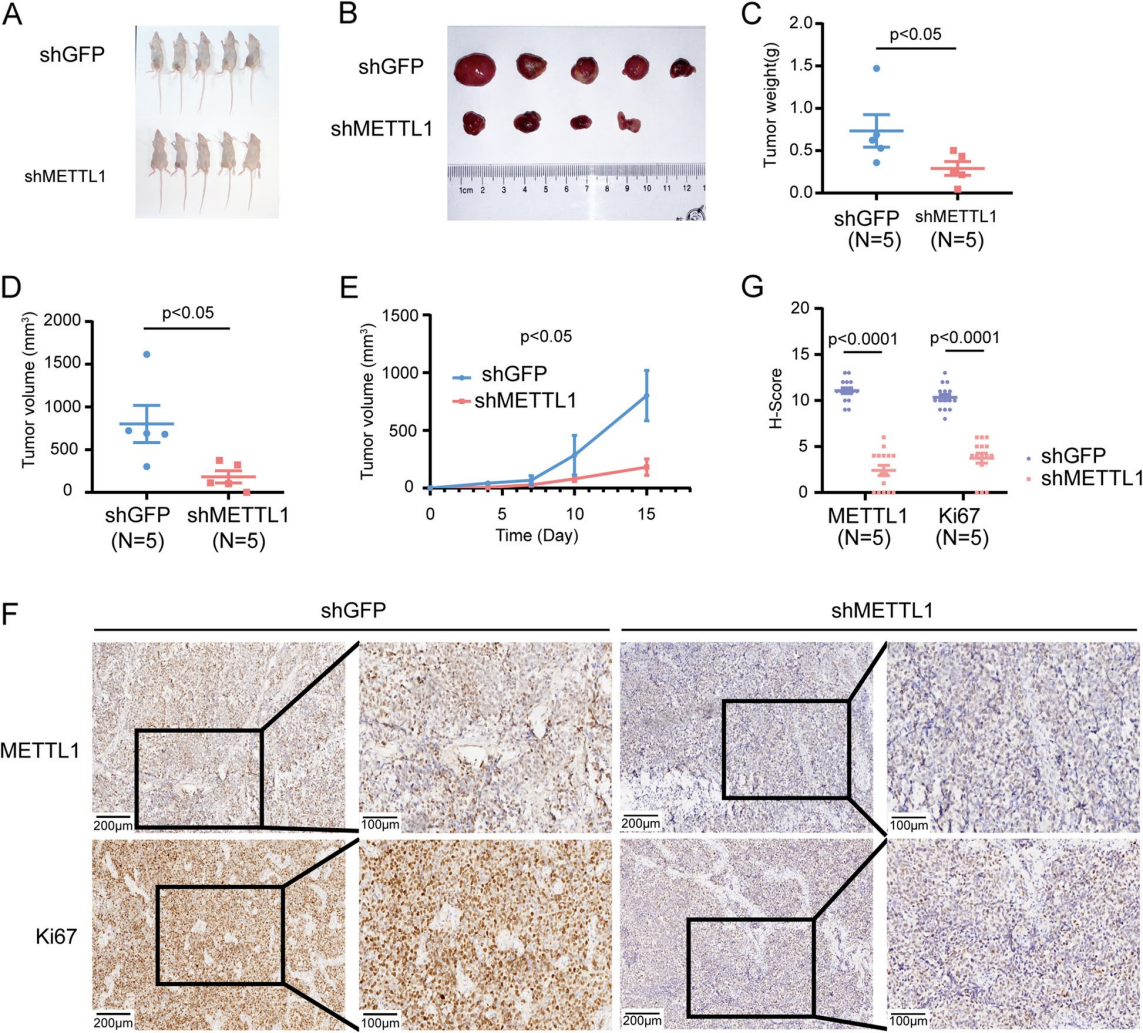
Inhibition of METTL1 reduces the tumorigenicity of NBL cells in vivo. **A**, **B** Tumor profiles of mice implanted with METTL1 knockdown and control NBL cells (*N* = 5). **C**, **D** Quantification of tumor weight (**C**) and size (**D**) at endpoints. **E** Growth curves of tumor volumes in the METTL1 knockdown and control groups. **F** Representative images of METTL1 and Ki67 IHC staining. **G** Quantitative analysis of IHC scores for METTL1 and Ki67. Data are presented as Mean ± SEM. *P* values were calculated by Mann-Whitney U test

We then used a xenograft mouse model to verify whether aberrant METTL1 expression affects NBL formation and growth in vivo. The tumorigenicity of mice injected with METTL1-knockdown BE2C cells was suppressed compared to controls, as reflected by a significant decrease in tumor size and weight (Fig. 4A-E). IHC staining of subcutaneous tumor tissues showed significantly reduced protein levels of METTL1 and Ki67, indicating that METTL1 knockdown significantly inhibited NBL proliferation in vivo (Fig. 4F-G).

### METTL1 regulated tRNA m^7^G modification, tRNA expression, and overall mRNA translation in NBL cells

**Fig. 5:**
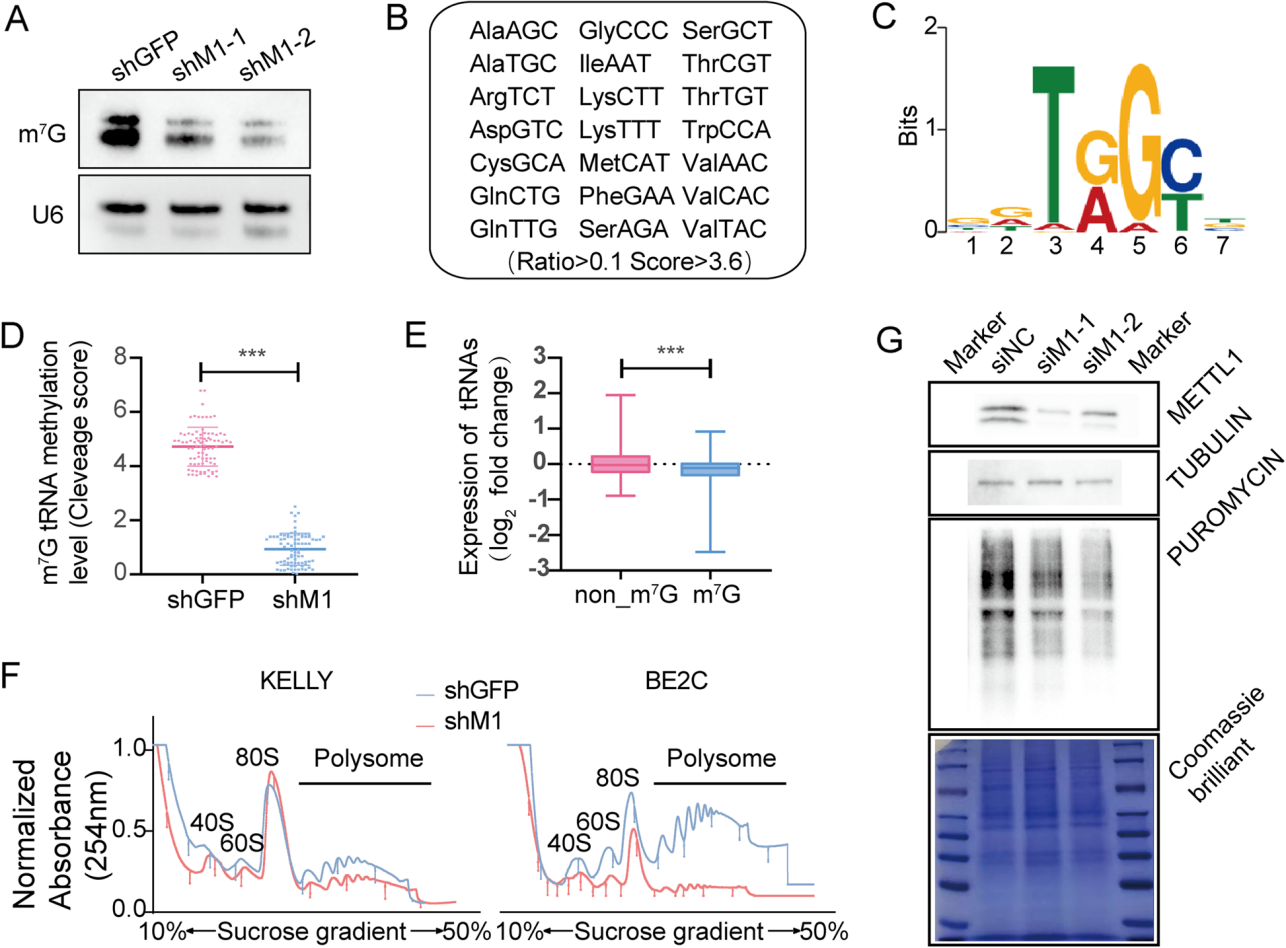
METTL1 regulates tRNA m^7^G modification, tRNA expression and global mRNA translation in NBL cells. **A** Northwestern blot confirmed decreased levels of m^7^G modification in METTL1-knockdown NBL cells (KELLY shM1–1). U6 snRNA was used as a loading control. **B** A total of 21 m^7^G -modified tRNAs were identified by TRAC-seq. **C** Motif sequences at the tRNA m^7^G site. **D** Quantification of m^7^G-modified tRNAs in METTL1 knockdown and control cells (KELLY shM1–1 and KELLY shNC). **E** Knockdown of METTL1 in NBL cells resulted in reduced expression of m^7^G -modified tRNAs. **F** Polysome profiling of METTL1 knockdown and control NBL cells. **G** Puromycin intake assay of METTL1-inhibited and control KELLY cells. Data are expressed as Mean ± SD. *P* values were calculated by Mann-Whitney U test

We explored the oncogenic function of METTL1 in regulating NBL progression in vivo and in vitro. To explore the molecular mechanisms underlying METTL1-mediated regulation of NBL progression, northwestern blot and northern blot were first performed, and we found a dramatic decrease in the level of m^7^G modification in METTL1-knockdown KELLY cells (Fig. 5A). We determined the tRNA m^7^G modification profile using previously established tRNA m^7^G site reduction and cleavage sequencing (TRAC-seq) (Lin et al., 2019), by which we identified a total of 21 m^7^G -modified tRNAs and the corresponding motif sequence “DURGY” (Fig. 5B-C). The m^7^G signal in METTL1 knockdown KELLY cells was significantly lower than in control cells (Fig. 5D). In addition, METTL1 knockdown resulted in a significant decrease in the expression level of m^7^G -modified tRNA in KELLY cells (Fig. 5D, E), indicating that METTL1-mediated tRNA m^7^G modification played a key role in regulating tRNA expression.

The tRNA is one of the key components in the mRNA translation process, so we performed a polysome analysis to assess the global translation level of METTL1 knockdown NBL cells. METTL1 knockdown inhibited translational activity compared to the control, which was reflected in reducing polysomalpeaks (Fig. 5F). In addition, the decrease in mRNA translation levels in METTL1 knockdown NBL cells was lso confirmed by the puromycin intake assay, as reflected in the reduction of incorporated protein (Fig. 5G). Altogether, our data demonstrated that METTL1-mediated tRNA m^7^G modification plays a vital role in controlling tRNA expression and mRNA translation in NBL cells.

### Knockdown of METTL1 selectively inhibited the translation of oncogenic mRNAs

**Fig. 6:**
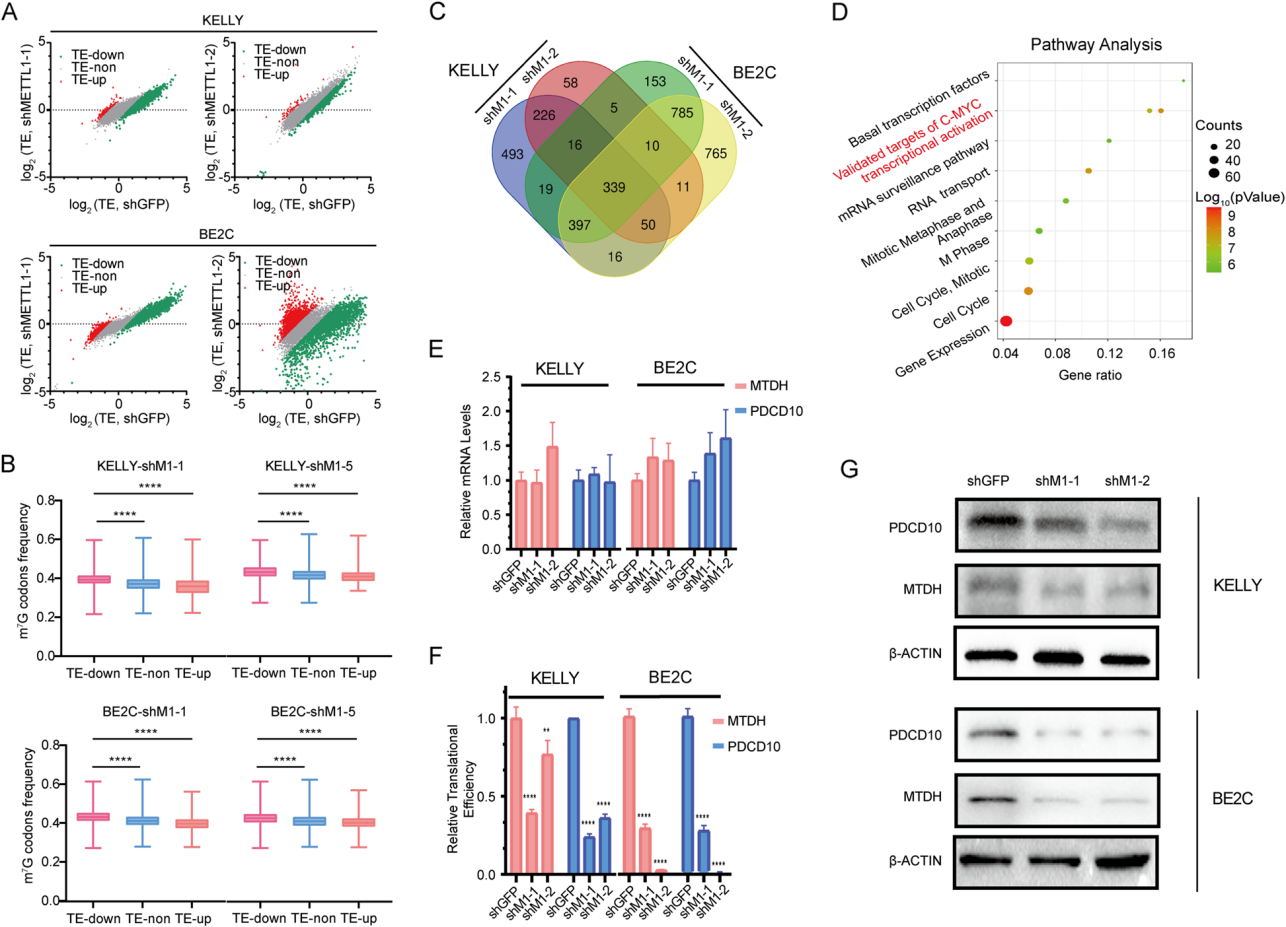
Knockdown of METTL1 selectively inhibits translation of oncogenic mRNAs. **A** TE Scatter plot in METTL1 knockdown and control NBL cells. **B** Frequency of m^7^G tRNA decoding codons for increased TEs (TE-up), decreased TEs (TE-downdown), and unaltered TEs (TE-non). **C** Venn diagram of genes with decreased TEs in KELLY and BE2C cells. **D** Pathway enrichment of TE-down genes. **E** mRNA levels of MTDH and PDCD10. **F** RNC-qPCR confirmed translational downregulation of MTDH and PDCD10 after METTL1 knockdown. **G** Western blot confirmed the decreased protein levels of MTDH and PDCD10 in METTL1 knockdown NBL cells. Data are presented as Mean ± SD. *P values* were calculated by One-way ANOVA with Dunnett’s multiple comparisons test for (**B**, compared to TE-Down group), (**E**-**F**, compared to the shGFP group). **p < 0.01, ****p < 0.0001

To further study the translational mechanism of METTL1-mediated tRNA m^7^G modifications in NBL progression, we sequenced actively translated mRNAs using ribosome nascent-chain complex sequencing (RNC-seq). mRNAs bound to ribosome nascent-chain complex (RNC-mRNAs) were isolated from total mRNAs (input-mRNAs) by centrifugation. The TE of mRNA was calculated by dividing the FPKM of input-mRNAs by the FPKM of RNC-mRNA. mRNAs with down-regulated TE (TE-down) or upregulated TE (TE-up) were identified by RNC-seq (Fig. 6A). To investigate the association between down-regulated m^7^G-modified tRNA expression and TE-down mRNAs, we calculated the frequency of m^7^G-modified tRNA-decoded codons on all mRNAs. Interestingly, the frequency of the codons decoded by m^7^G -modified tRNAs on TE-down mRNAs was significantly higher than that of other mRNAs, suggesting that METTL1-mediated tRNA m^7^G modification selectively regulates mRNA translation in the form of m^7^G -related codon dependent manner (Fig. 6B).

We next took the intersection of TE-down mRNAs in KELLY and BE2C cells and found 339 candidates (Fig. 6C). Gene ontology analysis of candidate mRNAs showed that TE-down mRNAs in METTL1 knockdown cells were significantly enriched in oncogenic pathways, including the genomes of c-MYC transcriptional activation and validated targets of the cell cycle (Fig. 6D). Besides, the expressions of the mRNAs in validated targets of c-MYC transcriptional activation pathway, were not significantly changed or even up-regulated (Supplementary Fig. 2A-D). That means the decreased TE of validated targets of c-MYC transcriptional activation were not caused by mRNA, but depened on the m^7^G codons frequency. After comprehensive analysis of this powerful ontological pathway by combining with gene function,the level of m^7^G codons frequency and the reduced TE, Metadherin (MTDH, also known as AEG-1 and Lyric) and Programmed Cell Death 10 (PDCD10), two common oncogenes with a higher level of m^7^G codons frequency (Supplementary Fig. 2E) and significantly reduced TE(Supplementary Table 6) in METTL1 knockdown NBL cells, were selected as candidate genes. Intererstingly, he mRNA level, translation level and protein level of the candidate genes were verified by qPCR, RNC-qPCR and Western blot, and the results confirmed that downregulation of METTL1 in NBL cells significantly suppressed the expression of MTDH and PDCD10 at the translation level rather than the transcription level(Fig. 6E-G). Consistent with our RNC-seq data, our RNC-qPCR and Western blot data indicated that METTL1-mediated tRNA m^7^G modification regulated oncogenic expression by interfering with mRNA translation. Overall, our findings uncovered a selective regulatory function of METTL1 in oncogenic mRNA translation and revealed a molecular mechanism for tRNA m^7^G modification mediated NBL progression.

## Discussion

Epigenetic plasticity plays a vital role in regulating the phenotype of tumor cells [38, 39]. Notably, aberrant expression of the m^7^G -modified methyltransferase METTL1 was associated with a range of cancers. The potential role of METTL1 in catalyzing m^7^G modifications is different in different cancers [19, 40, 41]. For example, METTL1 exerts oncogenic activity via suppression of PTEN signaling in hepatocellular carcinoma and via the AKT/mTORC1 pathway in lung adenocarcinoma [19, 42]. In this study, we demonstrated for the first time the oncogenic role of METTL1-mediated tRNA m^7^G modification in NBL. Higher METTL1 expression is an novel independ risk factor beyond INSS stage, age, MYCN status and COG risk classification and confers a survival disadvantage to NBL. Furthermore, in vitro and in vivo experiments confirmed that knockdown of METTL1 expression inhibited tumorigenic capacity, cell activity, and migration ability of NBL and promoted apoptosis of cells. Therefore, METTL1 knockdown can inhibit the progression of NBL in vitro and in vivo.

In our study, METTL1-mediated tRNA m^7^G modification was shown to selectively regulate the translation of oncogenic transcripts in a codon-dependent manner, indicating METTL1-mediated NBL progression. mRNA translation is closely coordinated by mRNA, tRNA, and ribosomes and is a key link in transmitting genetic information from DNA to proteins. Previous studies have illustrated that dysregulation of mRNA translation encoding oncogenes leads to aberrant expression of oncogenes [43–46]. Therefore, targeting mRNA translation is a vital strategy to regulate gene expression.

To date, m^7^G modification is often located at the 5′ caps and internal positions of eukaryotic mRNA or internally within rRNA and tRNA of all species. Emerging evidences have uncovered that m^7^G methylation also occurs in internal mRNAs. In mammals, METTL1 catalyzes the formation of m^7^G at position 46 in tRNA, internal mRNAs and miRNAs. However, a recent paper argued that there is no m^7^G modification on miRNAs [47]. Like miRNAs, m^7^G in mRNAs need to be proved in the following research because of its poorly understanding by its low abundance and a lacking of sensitive-convenient detection. Given that, we had calculated the frequency of m7G-modified tRNA-decoded codons on all mRNAs and clearly found the frequency of the codons decoded by m^7^G-modified tRNAs on TE-down mRNAs was significantly higher than that of other mRNAs. Therefore, the decrease of m^7^G-tRNAs upon METTL1 knockdown cannot completely attribute to the inhibition of mRNA translation mentioned in this study, but at least it can be considered to have contributed to them.

A growing body of evidence reveals the oncogenic functions of MTDH and PDCD10 in regulating cancer progression. For example, MTDH has been reported to affect the function of PI3K/AKT pathway genes directly or indirectly and is associated with tumor cell survival, metastasis, and drug resistance [48–55]. In addition, a study showed that knockdown of MTDH in human NBL significantly inhibited cell survival and significantly improved sensitivity to cisplatin [56]. PDCD10 promotes cell migration and tumor metastasis through epithelial-mesenchymal transition and the Wnt signaling pathway and is involved in apoptosis and cell cycle regulation [57, 58]. These studies provided direct evidence for targeting MTDH and PDCD10 for better cancer management. In the present study, we found that METTL1 and its downstream MTDH and PDCD10 have oncogenic functions in the progression and occurrence of NBL, suggesting that METTL1 or its downstream MTDH and PDCD10 may be potential targets for NBL therapy. Unfortunately, we did not perform downstream gene rescue experiments, and further validation is needed.

## Conclusion

In conclusion, enhanced expression of METTL1 in patients predicts a poor prognosis. Furthermore, METTL1 knockdown reduces m^7^G tRNA modification and selectively reduces mRNA translation of oncogenes in NBL in a codon frequency-dependent manner, which could explain why METTL1 knockdown inhibits tumorigenesis and progression in NBL cells in vitro and in vivo. The results of this study enrich the network of epigenetics in the regulation of neuroblastoma progression, and METTL1 is expected to provide new biomarkers and novel therapeutic targets for the diagnosis of NBL, significantly advanced NBL.

## Supplementary Information

**Additional file 1.**

**Additional file 2.**

**Additional file 3.**

## Data Availability

The raw data are available on reasonable request from corresponding author.

## Abbreviations

NBL: Neuroblastoma
EFS: Event-free survival
m^7^G: N7-methylguanosine
METTL1: Methyltransferase-like 1
GEO: Gene Expression Omnibus
IHC: Immunohistochemistry assay
TRAC-seq: tRNA reduction and cleavage sequencing
RNC-seq: Ribosome nascent-chain complex-bound mRNA sequencing
TE: Translational efficiencies
ncRNA: Non-coding RNA
lncRNAs: Long non-coding RNAs
miRNAs: MicroRNAs
MYCN: MYCN proto-oncogene
m^6^A: N6-methyladenosine
WDR4: WD repeat domain 4
ARRIVE: Animal Research: Reporting of in vivo Experiments
HEK 293 T: Human embryonic kidney 293 T
BE2C: Neuroblastoma cell line: SK-N-BE (2) C
DMEM: Dulbecco’s Modified Eagle Medium
FBS: Fetal bovine serum
DMEM/F-12: Dulbecco’s Modified Eagle Medium/Nutrient Mixture F-12
shRNA: Short hairpin RNA
siRNA: Small interfering RNA
3′ UTR: 3′ untranslated regions
shGFP: shRNA targeting green fluorescent protein
shM1: shRNA targeting METTL1
PBS: Phosphate-buffered saline
H-score: Histochemistry score
AG: AG RNAex Pro RNA Reagent
RT-PCR: Reverse transcription-polymerase chain reaction
qPCR: Real-time quantitative PCR assays
EDTA: Ethylene Diamine Tetraacetic Acid
snRNA: Small nuclear RNA
MOPS: 3- (N-morpholino) propanesulfonic acid
PMSF: Phenylmethylsulfonyl Fluoride
OD: Optical density
siNC: siRNA targeting negative control oligos
siM1: siRNA targeting METTL1
TRAC-seq: tRNA m7G reduction and cleavage sequencing
RNC-seq: Ribosome nascent-chain complex-bound mRNA sequencing
RB buffer: Ribosomal buffer
FPKM: Fragments per kilobase per million
TE-down: Down-regulated Translational efficiencies
COG: Children’s Oncology Group
INSS: International Neuroblastoma Staging System
RNC-mRNAs: mRNAs bound to ribosome nascent-chain complex
Input-mRNAs: Total mRNAs
TE-up: Upregulated Translational efficiencies
TE-non: No significant differences in Translational efficiencies
MTDH: Metadherin
PDCD10: Programmed Cell Death 10
