# Supplementary material for: METTL1 promotes neuroblastoma development through m7G tRNA modification and selective oncogenic gene translation
Source: Biomark Res. 2022 Sep 7;10:68. doi: 10.1186/s40364-022-00414-z (PMC9454133; doi:10.1186/s40364-022-00414-z)
Supplement: Supplementary file 1 — Additional file 1. [file 40364_2022_414_MOESM1_ESM.docx]

**
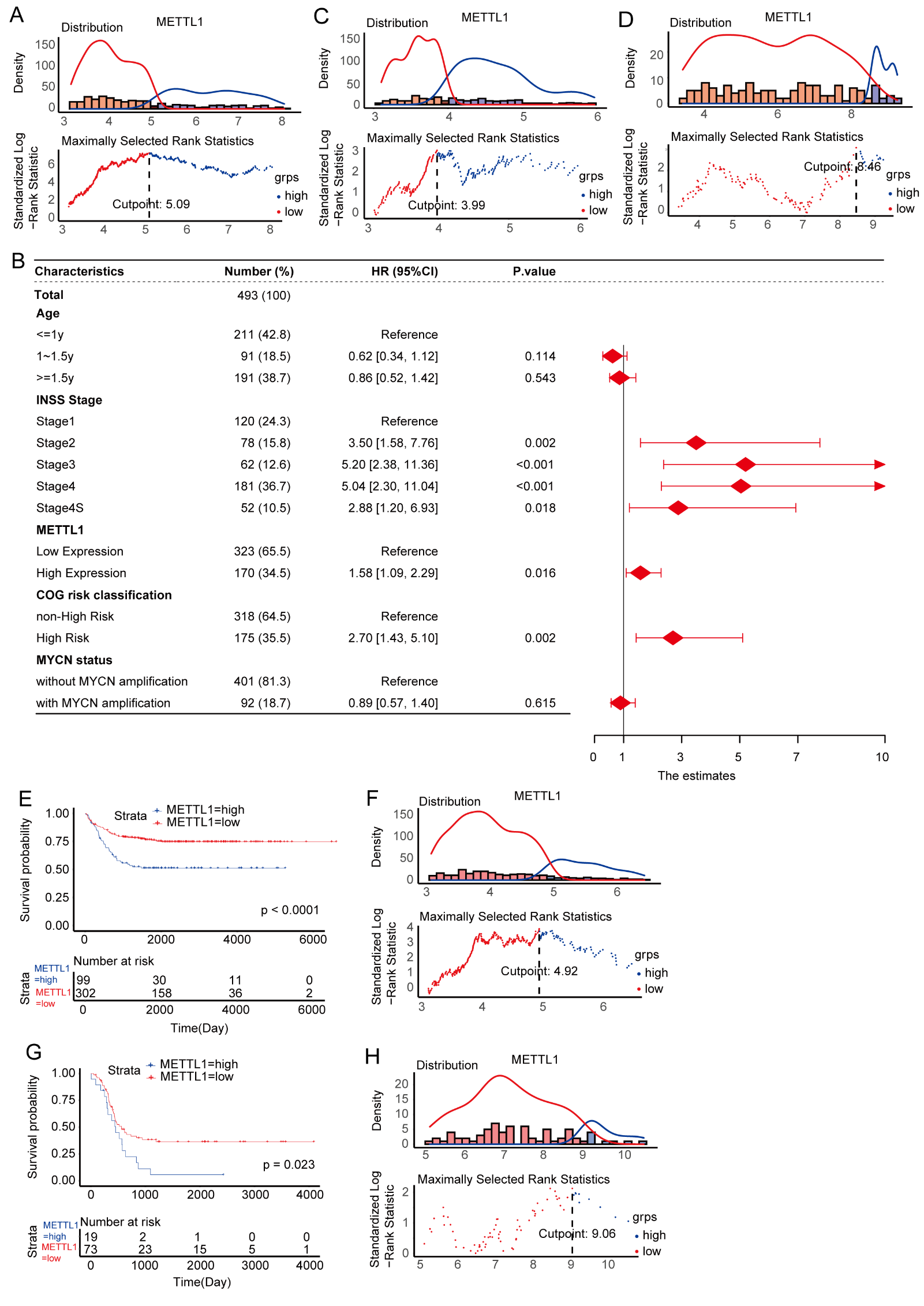
**

**Supplementary Figure 1. The clinical association between METTL1 and NBL in GEO datasets (GSE62564).** (A) The determination of the optimal cutoff of METTL1 expression levels among a total of 498 NBL patients. (B) Forest plot of multivariate Cox regression analysis among 493 patients with MYCN status records in GEO datasets, in which the hazard ratio (HR), 95% confidence interval (95% CI) and p values were denoted. METTL1 expression levels were grouped according to the optimal cutoff shown in the Supplementary Figure 1A. (C-D) The optimal cutoff of METTL1 expression levels among 322 of 498 with non-high risk NBL(C) and 176 of 498 with high risk NBL(D). (E-F) Kaplan-Meier analysis of the event-free survival of patients without MYCN amplification based on METTL1 expression levels (E, N= 401) and its cutoff was shown clearly(F). (G-H) Kaplan-Meier analysis of the event-free survival of patients with MYCN amplification based on METTL1 expression levels (G, N= 92) and its cutoff was also shown precisely (H). The whole cutoff values were determined by R Package *survminer* *(https://cran.r-project.org/web/packages/survminer/index.html)*. *P* values were calculated by Log-rank test.

**
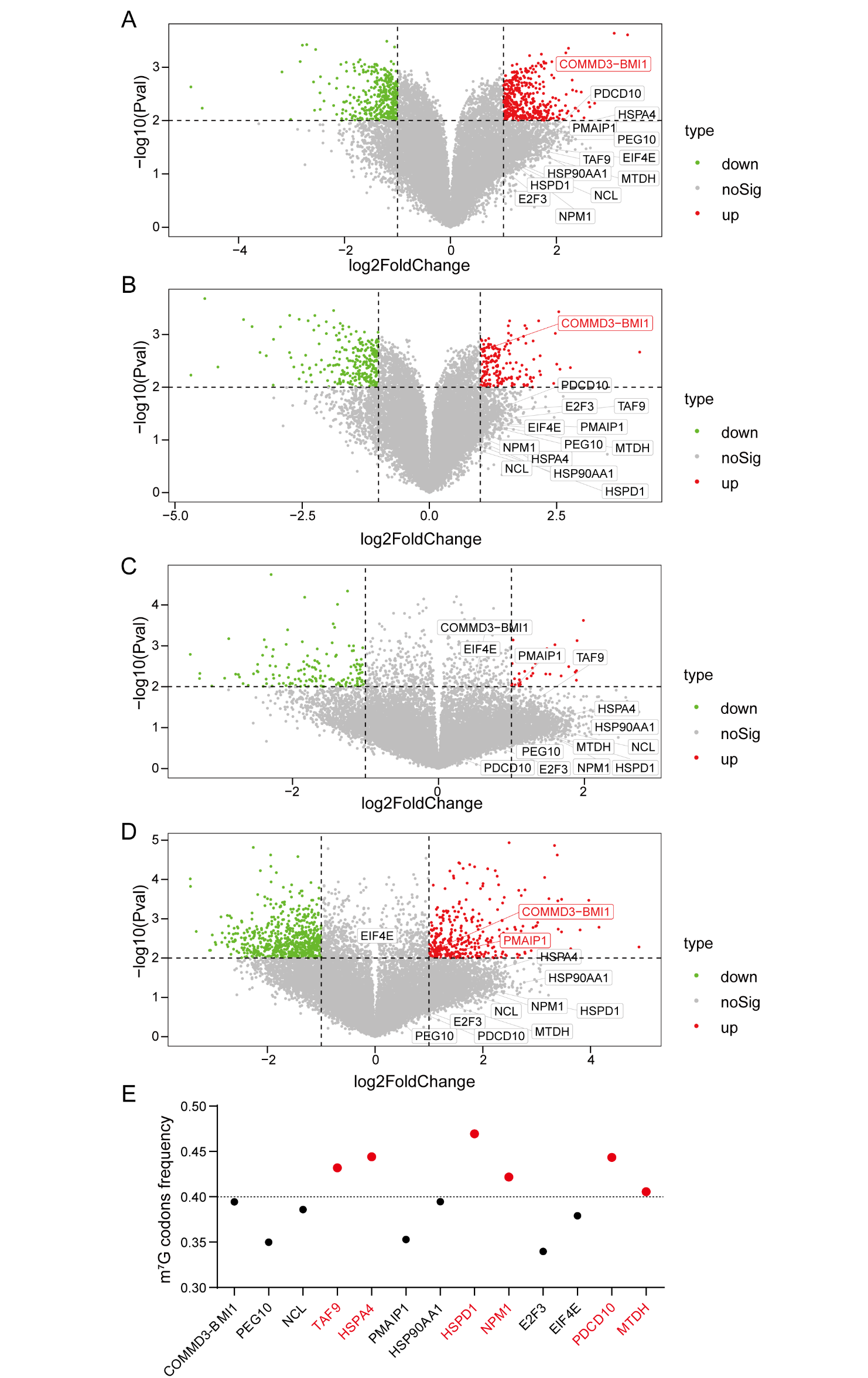
**

**Supplementary Figure 2.** **Further analysis of RNC-seq data.** (A-D) Volcano plots were used to show the expression level of each mRNAs in KELLY shM1-1(A), KELLY shM1-2 (B), BE2C shM1-1(C) and BE2C shM1-2(D). X-axis: the fold change expressed as log2; Y-axis: p value expressed as -log10; The horizontal dotted line in the middle represented a p value of 0.01; The two vertical dashed lines represented a log2FoldChange of -1(left) and 1(right); The points in the plots represented mRNAs that were down-regulated (‘down’, marked in green) up-regulated (‘up’, marked in red) and expressed no differentially ('noSig’, marked in black) with statistical difference. The validated targets of c-MYC transcriptional activation were labeled. (E) Frequency of m^7^G tRNA decoding codons for the validated targets of c-MYC transcriptional activation, in which those with higher level frequency were highlighted in red.

**Supplementary Table 1. Baseline information of 132 patients with neuroblastoma.**

| **Characteristic** | **Number** | | |
| --- | --- | --- | --- |
|  | **Total** | **High H-Score** | **Low H-Score** |
| **INSS Stage** | 132 | 74 | 58 |
| Stage1 | 20 | 0 | 20 |
| Stage2 | 19 | 0 | 19 |
| Stage3 | 17 | 9 | 8 |
| Stage4 | 76 | 65 | 11 |
| **Characteristic** | **Number** | **Median** | **Mean±SEM** |
| H-Score | 132 | 6 | 6.15±0.31 |

**Supplementary Table 2. Baseline information of 498 patients with neuroblastoma in the GEO datasets**

| **Characteristic** | **Number** | **Percentage (%)** |
| --- | --- | --- |
| **Age** |  |  |
| <=1y | 212 | 42.57 |
| 1-1.5y | 93 | 18.67 |
| >=1.5y | 193 | 38.76 |
| **INSS Stage** |  |  |
| Stage1 | 121 | 24.30 |
| Stage2 | 78 | 15.66 |
| Stage3 | 63 | 12.65 |
| Stage4 | 183 | 36.75 |
| Stage4S | 53 | 10.64 |
| **Gender** |  |  |
| Male | 287 | 57.63 |
| Female | 211 | 42.37 |
| **COG risk classification** |  |  |
| Non-High Risk | 322 | 64.66 |
| High Risk | 176 | 35.34 |
| **MYCN status** |  |  |
| without MYCN amplification | 401 | 80.52 |
| with MYCN amplification | 92 | 18.47 |
| NA | 5 | 1.00 |

NA means there is no record of MYCN status.

**Supplementary Table 3. Oligonucleotides sequences**

| **Oligonucleotides** | **Sequence (5’-3’)** |
| --- | --- |
| **The sequences of METTL1 shRNA** |  |
| shM1-1 | AAATGAGTGCACATCCAGTCG |
| shM1-2 | TTTCTTATCCTTTGGGTCATC |
| **The sequences of METTL siRNA** |  |
| siM1-1 | GATGACCCAAAGGATAAGAAA |
| siM1-2 | GGATGTGCACTCATTTCGA |
| **Probe sequences of tRNAs and U6 snoRNA** |  |
| U6 snoRNA | TGGAACGCTTCACGAATTTG |
| **Primers used for the qRT-PCR assay** |  |
| hPDCD10-F | GCCCCTCTATGCAGTCATGTA |
| hPDCD10-R | AGCCTTGATGAAAGCGGCTC |
| hMTDH-F | GGAGTCAAGACACTGGAGATGC |
| hMTDH-R | GGGTTGATTACGGCTAACATCC |
| hβ-ACTIN-F | CACCATTGGCAATGAGCGGTTC |
| hβ-ACTIN-R | AGGTCTTTGCGGATGTCCACGT |

**Supplementary Table 4. Antibodies used in this study**

| **Antibody** | **Source** | **Cat No.** |
| --- | --- | --- |
| Rabbit polyclonal anti-METTL1 | Proteintech | Cat# 14994-1-AP |
| Rabbit polyclonal anti-Ki67 | Proteintech | Cat# 27309-1-AP |
| Mouse monoclonal beta ACTIN | Synaptic Systems | Cat# 251011 |
| Rabbit polyclonal beta TUBLIN | Abcam | Cat# ab18207 |
| Mouse monoclonal anti-puromycin | Millipore | Cat# MABE343 |
| Anti-rabbit IgG HRP-linked Antibody | Cell signaling technology | Cat# 7074S |
| Anti-mouse IgG HRP-linked Antibody | Cell signaling technology | Cat# 7076S |
| Mouse monoclonal 7-methylguanosine (m^7^G) antibody | MBL International | Cat# RN017M |
| Anti-Digoxigenin-AP | Roche | Cat# 11093274910 |
| AEG-1/MTDH-specific polyclonal Antibody | Proteintech | Cat# |
| PDCD10 Monoclonal Antibody | Proteintech | Cat# |

**Supplementary Table 5.** **Univariate and multivariate cox hazard analysis of risk factors in GEO datasets (N=493)**

| **Variable** | **Univariate analysis** | | | **Multivariate analysis** | | |
| --- | --- | --- | --- | --- | --- | --- |
|  | *HR* | *95%CI* | *P* | *HR* | *95%CI* | *P* |
| **Age** |  |  |  |  |  |  |
| <=1y (N=211) | Reference | - | - | Reference | - | - |
| 1-1.5y (N=91) | 0.70 | 0.40-1.23 | 0.213 | 0.62 | 0.34-1.12 | 0.114 |
| >=1.5y (N=191) | 3.02 | 2.16-4.22 | **<0.001** | 0.86 | 0.52-1.42 | 0.543 |
| **INSS Stage** |  |  |  |  |  |  |
| stage1 (N=120) | Reference | - | - | Reference | - | - |
| stage2 (N=78) | 3.69 | 1.67-8.15 | **0.001** | 3.50 | 1.58-7.76 | **0.002** |
| stage3 (N=62) | 7.30 | 3.42-15.60 | **<0.001** | 5.20 | 2.38-11.36 | **<0.001** |
| stage4 (N=181) | 12.3 | 6.24-24.30 | **<0.001** | 5.04 | 2.30-11.04 | **<0.001** |
| stage4S (N=52) | 3.75 | 1.60-8.77 | **0.002** | 2.88 | 1.20-6.93 | **0.018** |
| **Gender** |  |  |  |  |  |  |
| Male (N=284) | Reference | - | - | - | - | **-** |
| Female (N=209) | 1.15 | 0.86-1.54 | 0.361 | - | - | **-** |
| **METTL1^#^** |  |  |  |  |  |  |
| Low Expression (N=323) | Reference | - | - | Reference | - | **-** |
| High Expression (N=170) | 2.92 | 2.18-3.93 | **<0.001** | 1.58 | 1.09-2.29 | **0.016** |
| **COG risk classification** |  |  |  |  |  |  |
| Non-High Risk (N=318) | Reference | - | **-** | Reference | - | **-** |
| High Risk (N=175) | 5.29 | 3.87-7.24 | **<0.001** | 2.70 | 1.43-5.10 | **0.002** |
| **MYCN status** |  |  |  |  |  |  |
| without MYCN amplification (N=401) | Reference | - | **-** | Reference | - | - |
| with MYCN amplification (N=92) | 3.22 | 2.35-4.41 | **<0.001** | 0.89 | 0.57-1.40 | 0.615 |

CI = confidence interval; HR = hazard ratio.

Values in bold signify *p<0.05.*

^#^ Gene expression levels were grouped according to the optimal cutoff shown in the Supplementary Figure 1A.

**Supplementary Table 6. Gene enriched in validated targets of C-MYC transcription activation pathway.**

| **Genes enriched in validated targets of C-MYC transcription activation pathway** |
| --- |
| HSPD1 |
| HSPA4 |
| PDCD10 |
| NPM1 |
| MTDH |
| HSP90AA1 |
| COMMD3-BMI1 |
| NCL |
| EIF4E |
| PMAIP1 |
| PEG10 |
| E2F3 |
| TAF9 |
