# Supplementary figures and images for: METTL1 promotes neuroblastoma development through m7G tRNA modification and selective oncogenic gene translation
Source: Biomark Res. 2022 Sep 7;10:68. doi: 10.1186/s40364-022-00414-z (PMC9454133; doi:10.1186/s40364-022-00414-z)

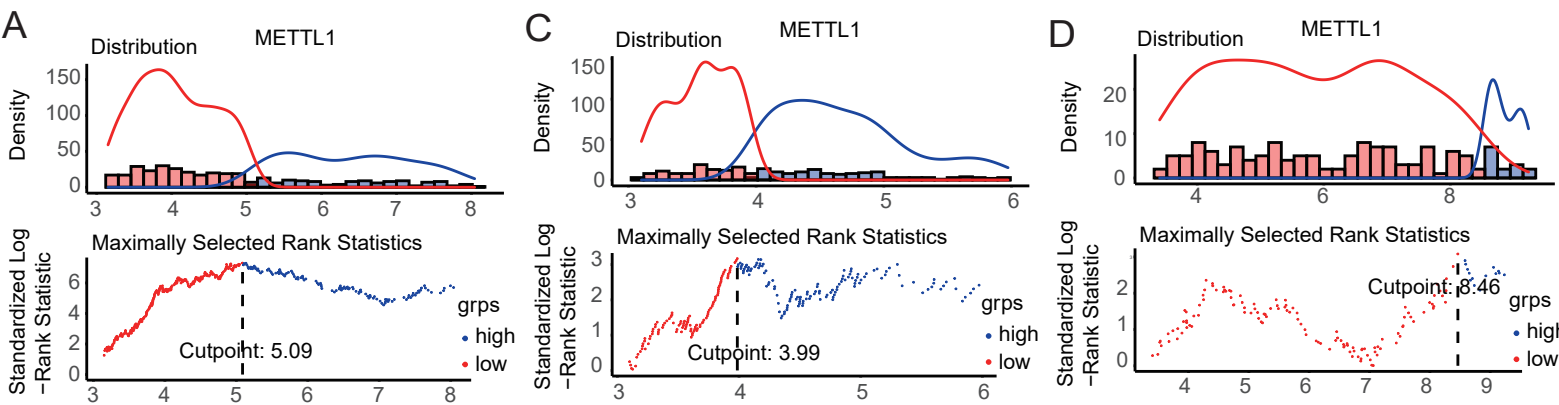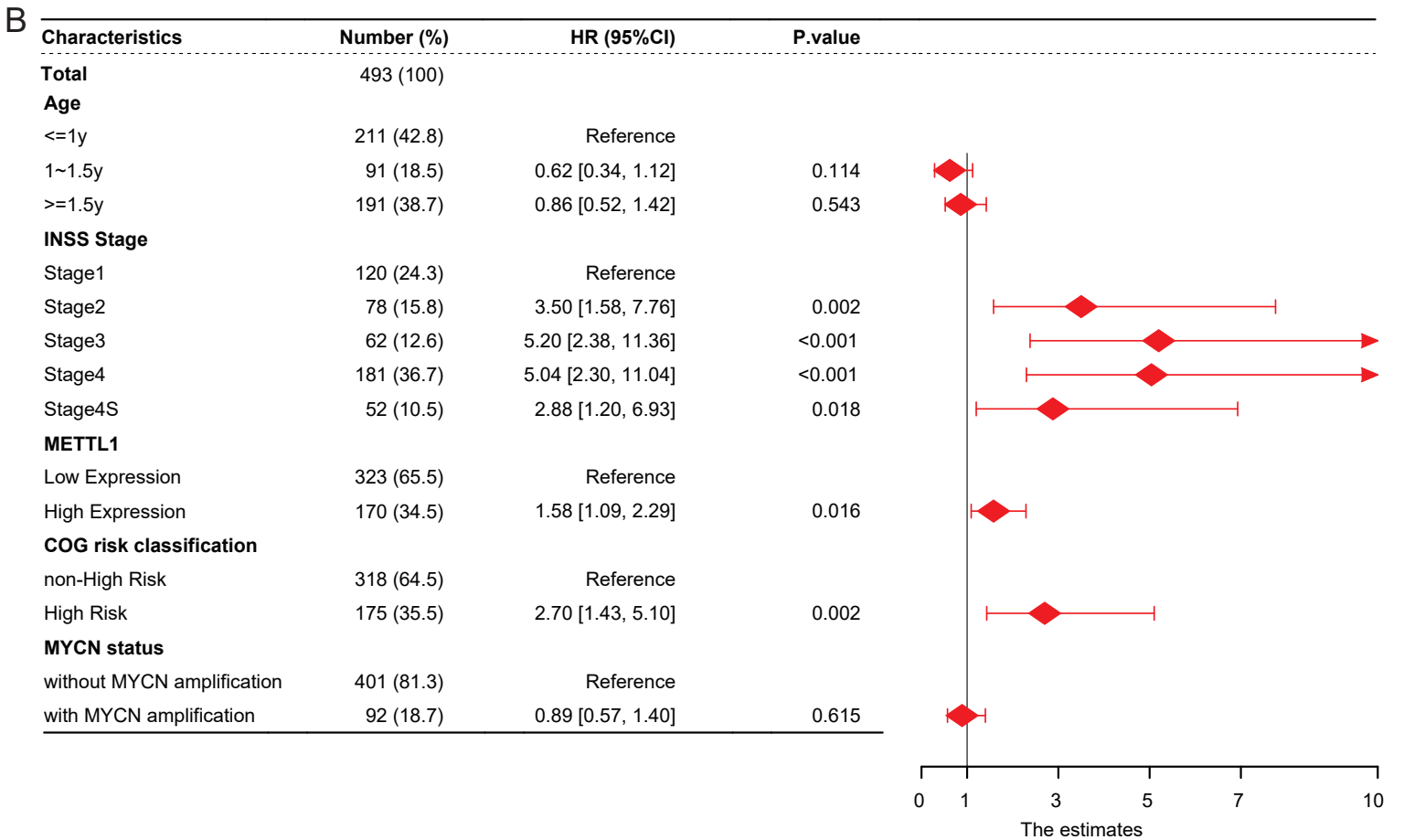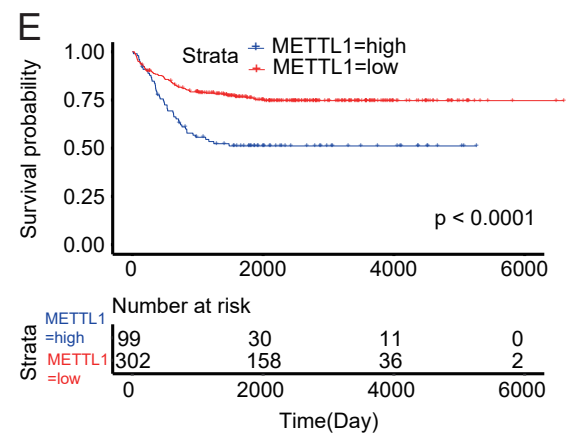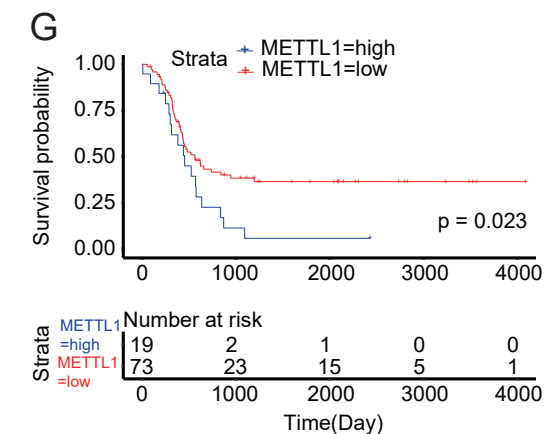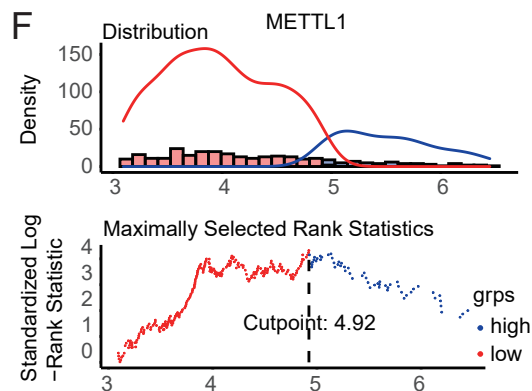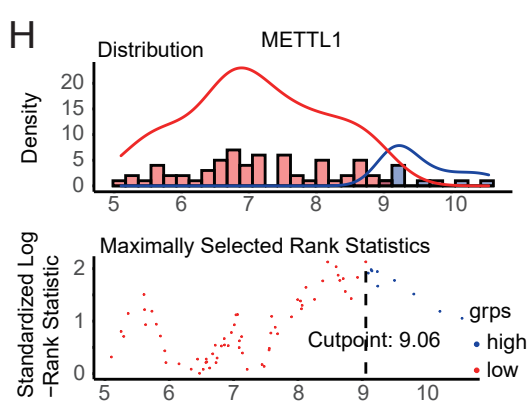

Supplement: Supplementary file 2 — Additional file 2. [file 40364_2022_414_MOESM2_ESM.pdf]

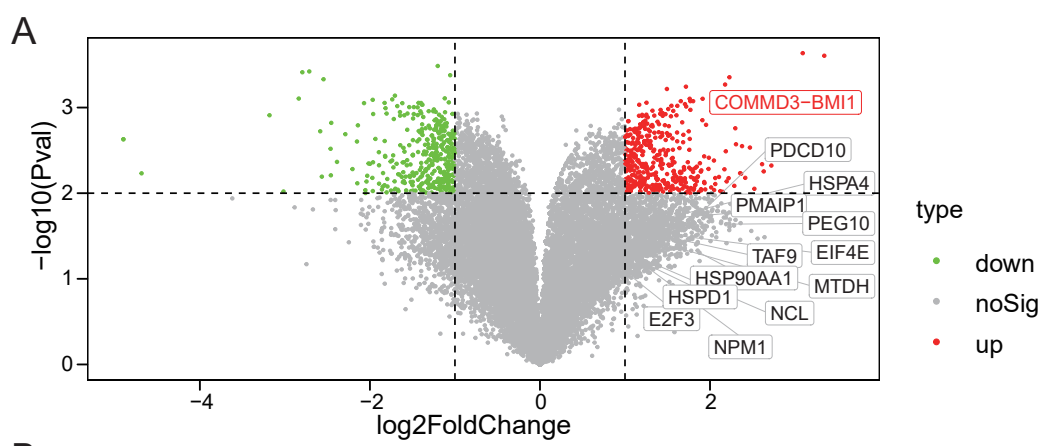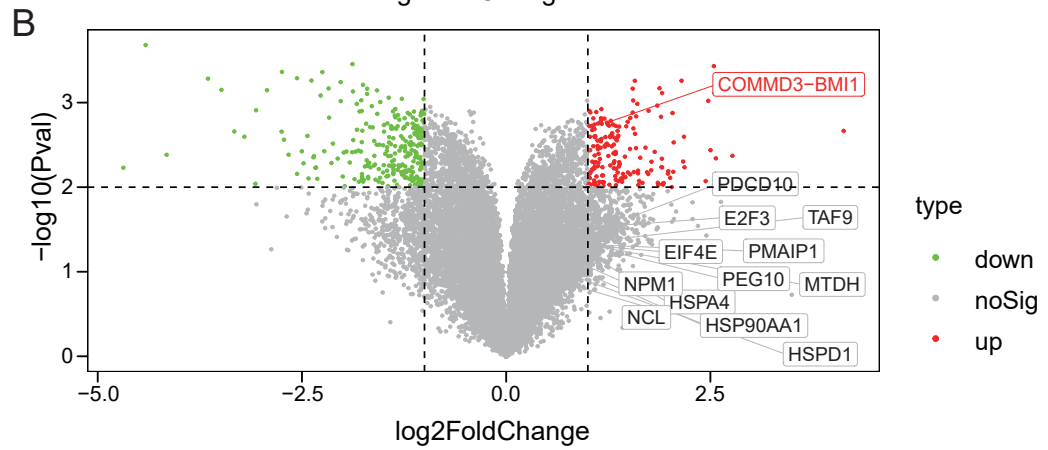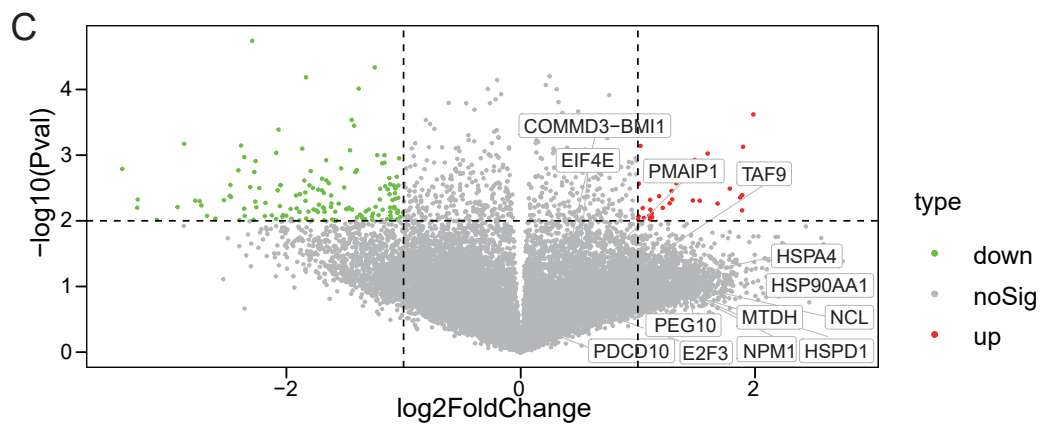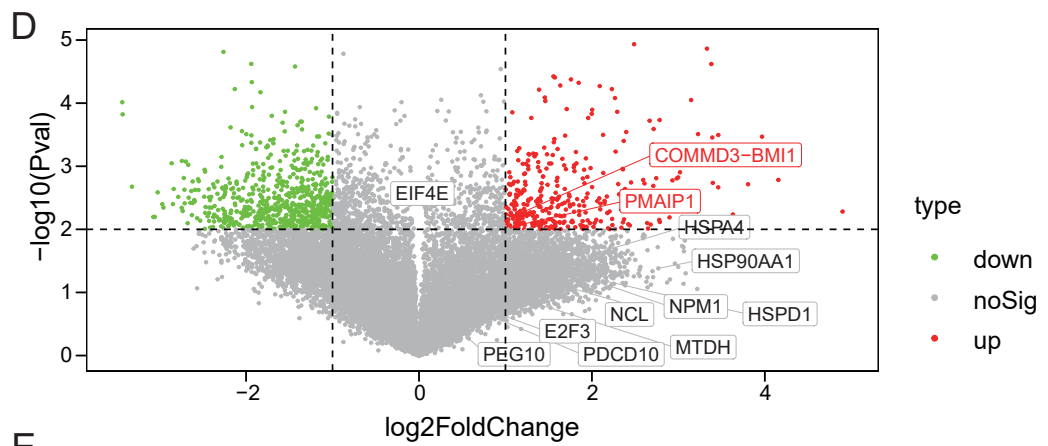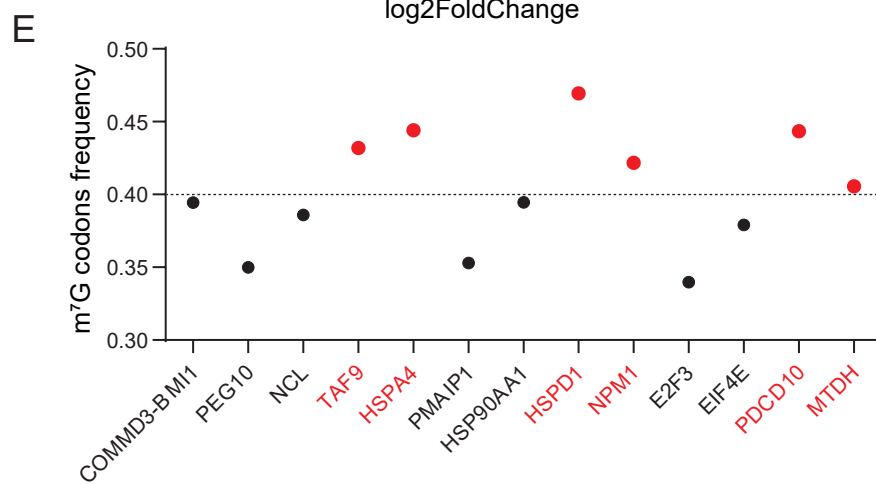

Supplement: Supplementary file 3 — Additional file 3. [file 40364_2022_414_MOESM3_ESM.pdf]
